# Aspergilloma in Non-tuberculous Cavities in the Lung: Not to Get Startled

**DOI:** 10.7759/cureus.27905

**Published:** 2022-08-11

**Authors:** Sai Tej Pavirala, S Anusha Rao, Gaurav Sahu, Alkesh Khurana, Abhishek Goyal

**Affiliations:** 1 Pulmonary and Critical Care Medicine, All India Institute of Medical Sciences, Bhopal, Bhopal, IND

**Keywords:** fungal ball, cavity, ipf, pulmonary embolism, aspergilloma

## Abstract

Aspergillus which is normally found as a colonizer in healthy individuals can manifest in various forms in patients with diseased lung or immunocompromised status. Aspergilloma is one such manifestation whereby the fungus makes its way into preexisting cavities in the lung, the most common underlying etiology being old tuberculous cavities, especially in countries with high TB prevalence. However, we hereby report two cases of Aspergillus infestation as aspergilloma in cavities because of extremely rare causes, namely pulmonary thromboembolism and idiopathic pulmonary fibrosis, respectively.

## Introduction

Aspergillus is a common fungus colonizing the pulmonary tissue in healthy individuals. However, in the diseased lung or immunocompromised status, this can cause varied manifestations, ranging from an incidentally detected aspergilloma and allergic bronchopulmonary aspergillosis to invasive aspergillosis. In countries with high TB prevalence, aspergilloma is known to occur in patients with pre-existing TB cavities. However, the occurrence of aspergilloma in cavities caused by other diseases is rare, e.g., in Wegner’s granulomatosis, sarcoidosis, etc. The two underlying etiologies, in this case, cavity secondary to pulmonary thromboembolism and Idiopathic pulmonary fibrosis (IPF) are even rarer causes and the clinician is very likely to miss them unless a high index of suspicion is kept in mind.

## Case presentation

Case report 1

**Figure 1:**
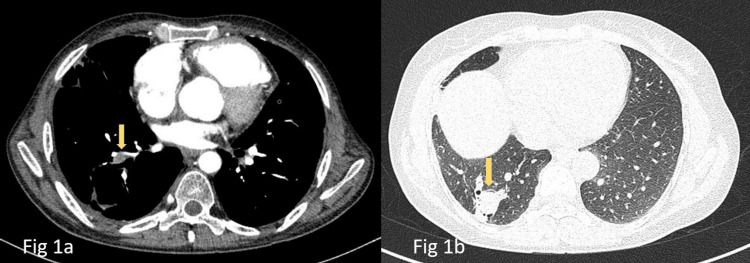
Aspergilloma in a patient of pulmonary thromboembolism (a) Computed tomography of the chest (mediastinal window) shows filling defect in the right basal pulmonary artery (downward arrow). (b) Computed tomography of the chest (parenchymal window) just a section below shows the same cavity with a meniscus sign suggestive of Aspergilloma (downward arrow).

A 45-year-old male, tobacco chewer with a previous history of poliomyelitis came with complaints of gradually progressing dyspnea, dry cough for the past one year and then gradually increasing pedal edema, abdominal distention for the past 10 days. On admission, he was hemodynamically stable, general physical examination revealed emaciated lower limbs due to poliomyelitis. Cardiovascular examination was suggestive of loud P 2 (second heart sound). Chest radiograph was suggestive of cardiomegaly, mild right-sided pleural effusion, and a right lower zone cavitary lesion. A 2D echocardiography showed significant dilatation of the right atrium and right ventricle with severe tricuspid regurgitation and pulmonary hypertension. However, left ventricular function was normal. As his pulmonary hypertension was out of proportion to the chest radiograph findings CTPA (CT Pulmonary Angiography) was planned. CTPA (Figure 1a) revealed a large eccentric hypodense filling defect partially filling the lumen of the right pulmonary artery accompanied by a partial filling defect in the right inferior lobar subsegmental arteries. Also, a thick-walled cavity with non-enhancing hyperdense content within its dependent portion was seen in the right superior and posterior basal segment of the right lower lobe in the same CT (Figure 1b). Considering the radiographic possibility of aspergillus infestation, Immunoglobulin G (IgG) specific for aspergillus was done and found to be positive. A final diagnosis of chronic pulmonary thromboembolism with chronic thromboembolic pulmonary hypertension (CTEPH) and Aspergilloma was made. Cavity with the fungal ball can very well be attributed to being secondary to the pulmonary infarct as there was no evidence of Pulmonary tuberculosis or any chronic pulmonary disease. The patient was started on Riociguat, anticoagulation, and diuretics and is currently doing well on follow-up.

Case report 2

**Figure 2:**
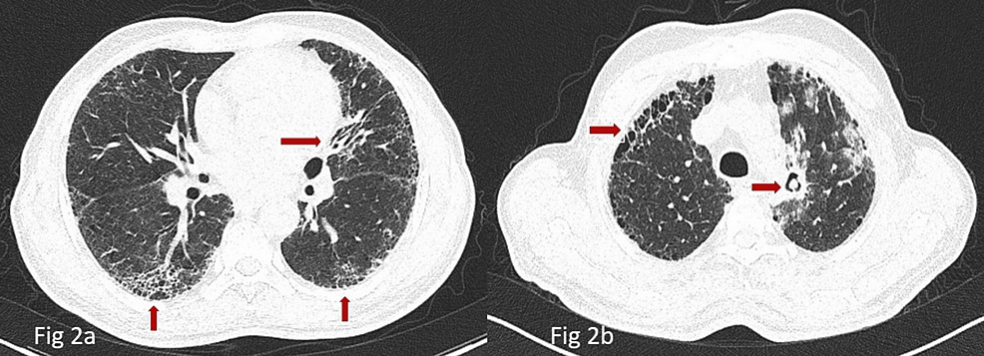
Aspergilloma in a patient of idiopathic pulmonary fibrosis (a) Computed tomography of the chest (parenchymal window) shows bilateral basal and subpleural honeycombing (Upward arrows) consistent with idiopathic pulmonary fibrosis. Also appreciable is tractional bronchiectasis in the left hilar region (horizontal arrow). (b) Computed tomography of the chest (parenchymal window) at a section above the carina shows a cavity in the left hilar region, apparently filled with a fungal ball highly suggestive of aspergilloma (right horizontal arrow). Also noticeable is subpleural honeycombing mixed with cystic air spaces in the periphery (left horizontal arrow).

A 67-year-old male, chronic smoker came with complaints of dry cough for the past two years and hemoptysis for the past four months. There was no past history of pulmonary tuberculosis or significant occupational history or exposure to pets. On examination, the patient had grade 2 clubbing, with stable vitals and basal fine inspiratory velcro crepitations. Bilateral reticular opacities were seen on the chest radiograph. High-resolution computed tomography (HRCT) was suggestive of bilateral inter and intralobular septal thickening with honeycombing along with interspersed ground glass opacities (GGOs) and a thick-walled cavity in the apico-posterior segment of the left upper lobe (Figures 2a, 2b, respectively). Computed tomography (CT) picture raised suspicion of aspergilloma. Sputum for AFB was negative and spirometry was inconclusive. IgG specific for aspergillus was done and was positive as in the previous case. Diffusion capacity of the lung for carbon monoxide (DLCO) was reduced (33%) and the six-minute walk distance was 490 m. Anti-nuclear antibodies were weakly positive (1:100) which was insignificant and extracted nuclear antigen (ENA) reflux, as well as rheumatoid factor, were negative. Thus the patient was labeled with IPF and coexisting aspergilloma.

## Discussion

Aspergillus can cause a wide spectrum of lung pathologies depending upon the host’s immune status and underlying lung condition. In normal healthy individuals, aspergillus can be a colonizer, or in individuals with atopy, it can manifest as allergic broncho pulmonary Aspergillosis (ABPA) or Aspergillus sinusitis. In individuals with decreased immunity, it can present as Invasive Aspergillosis. In pre-existing cavities, aspergillus can manifest as aspergilloma [1,2]. Aspergilloma is also known as mycetoma or fungal ball which consists of living and dead fungal elements, mucin, fibrin, cellular debris, blood elements, and inflammatory and epithelial cells [3]. The most common cause of aspergilloma in developing countries is post tubercular status which constitutes more than 95% of the cases. Other rare causes mentioned in the literature are sarcoidosis, asbestosis, cavitary neoplasia, bronchiectasis, lung abscess, and Wegener’s granulomatosis [4]. There are even rarer case reports mentioning its association with pulmonary infarction and IPF. We could find only two cases in literature whereby aspergilloma has been reported in association with IPF.

A clinico-radiological and serological confirmation is essential for the diagnosis of aspergilloma. The patient may present with hemoptysis or can be asymptomatic [5]. In our first case, the patient was asymptomatic and aspergilloma was an incidental radiological diagnosis when pulmonary embolism was being diagnosed and in the second case patient presented with hemoptysis, and CT was done to find out the etiology of hemoptysis. IPF was rather picked up incidentally in this case. Radiologically a well-defined cavity with a soft tissue density or heterogenous density within the cavity is seen and it is usually identified as an air crescent sign/air meniscus sign [6]. In both cases, we can identify the aspergilloma within the cavity with an air crescent sign. Serum IgG specific for aspergillus can be seen in about >95% of aspergilloma patients.

The mechanism of formation of aspergilloma in UIP is not known and not studied as there is a rare association between them, but Kumar et al. opined that it can be because of the chronic and debilitating nature of the disease and also because of the immunosuppressants used in its treatment [3]. Pulmonary infarction can lead to cavitation which is usually sterile but secondary infection with Gram-negative bacteria is also not uncommon. However, an infestation of the cavity with aspergillus and the formation of aspergilloma is rare.

Treatment of aspergilloma is mainly symptomatic by controlling hemoptysis. If hemoptysis is massive or fatal then bronchial artery embolization or surgical management is warranted [7]. But managing a patient with a debilitating disease like IPF and /or pulmonary embolism and aspergilloma is challenging. Antifibrotics like nintedanib and pirfenidone are the only available treatment agents for IPF whereas anticoagulants are the mainstay of treatment in pulmonary embolism. The need for anticoagulation in a patient with PTE and the presence of hemoptysis in the same patient because of aspergilloma may definitely perplex the treating physician. Some patients may also progress to chronic cavitary pulmonary aspergillosis (CCPA) when the role of azoles comes into play in the management. In these patients, one should consider the drug interactions before starting the management. Metabolism of antifibrotics and anticoagulants is strongly influenced by cytochrome P450 3A4 (CYP3A4) and CYP1A2 [8,9]. Antifungal azoles like itraconazole, and voriconazole which is the treatment of choice in CCPA are inhibitors of CYP3A4, and CYP1A2 which may lead to decreased metabolism of antifibrotics and anticoagulants causing increased side effects [9].

## Conclusions

Pulmonary embolism can have varied complications but Aspergilloma is certainly not the one commonly expected. Hence, it becomes prudent to diagnose this early, as anticoagulation, which is the therapeutic cornerstone of pulmonary embolism may increase the chances of hemoptysis. This calls for strict monitoring protocols in expert hands. Both of these requires different lines of management and hence high suspicion index is needed for its timely diagnosis and appropriate management. Similarly, coexistence of UIP and aspergilloma is very sparse. Whenever a case of UIP/ILD is presenting with hemoptysis one should have a high degree of suspicion to look for aspergilloma.
